# Tailoring the strength and porosity of rapid-hardening magnesia phosphate paste via the pre-foaming method

**DOI:** 10.1038/srep13016

**Published:** 2015-08-13

**Authors:** Li-Jie Liu, Jin-Hong Li, Xiang Wang, Ting-Ting Qian, Xiao-Hui Li

**Affiliations:** 1 Beijing Key Laboratory of Materials Utilization of Nonmetallic Minerals and Solid Wastes, National Laboratory of Mineral Materials, School of Materials Science and Technology, China University of Geosciences (Beijing), Beijing 100083, P.R. China

## Abstract

High-porosity magnesia phosphate paste (HPMPP) was prepared via the pre-foaming method. In the pre-foaming method, sintering treatment was not required. The bulk density and maximum compressive strength of the HPMPP prepared according to the ratio of water to solids (W/S_o_) of 0.32 reached 464.00 ± 5.00 Kg/m^3^ and 0.30 ± 0.05 MPa, respectively. The compressive strength increased with the increases in the addition amounts of sodium silicate and polypropylene fibers. The bulk density of HPMPP increased with the increase in the addition of sodium silicate and decreased with the increase in the addition of polypropylene fibers. Besides, the porosity of the magnesia phosphate paste increased from 79.85% to 81.27% and from 80.31% to 83.75% after the addition of sodium silicate and polypropylene fibers respectively. The highest porosity (83.75%) of the prepared HPMPP was realized under the addition proportion (sodium silicate: polypropylene fibers: solids = 0.06:0.0025:1). The average pore size of the prepared HPMPP is about 180 μm and the pore distribution range is relatively narrow. The hydration product (struvite) is combined with MgO particle one by one and then coated on the surface of bubbles. With the decrease of the water content, after breaking bubbles, the porous structure can be achieved.

As a cementitious material, cement has been the most widely used construction material since the utilization of flint in the Paleolithic times and has become an irreplaceable construction material with high strength and durability. The production of cement, however, generates numerous carbon dioxides and consumes a large quantity of electrical power and fuel energy, thus leading to serious environmental pollution^1^,^2^,^3^,^4^. Therefore, it is necessary to develop new cementitious materials from other resources.

The low-temperature treatment and stabilization technology was the key technology in the development of chemically bonded phosphate ceramics^5^. The most interesting property of the ceramics is that the feasible mechanical strength can be achieved without sintering. It is believed that magnesium phosphate cement (MPC) is one of the most promising candidates of this material. Traditional magnesium phosphate cement is synthesized via the chemical reaction with dead burned magnesium (MgO) and ammonium dihydrogen phosphate (NH_4_H_2_PO_4_)^6^. Traditional MPCs had been extensively investigated. However, most of the studies on MPCs were concentrated on their engineering properties and aimed to obtain a compact material that could be directly applied^7^,^8^,^9^. The feasible way to expand the applications of MPC-based materials is to develop new porous materials of magnesia phosphate with the similar structure to foam concrete. Actually, foam concrete is a kind of lightweight concrete which possesses high fluidity, low self-weight, minimal aggregate consumption, uniform porous structure, and excellent thermal insulation properties^10^,^11^,^12^. Moreover, the low strength of pores can be enhanced by magnesia phosphate cement with rapid hardening speed and early strength. Through the proper control of the dosage of foam, foamed concrete with a wide density range (1600–400 Kg/m^3^) can be obtained for the applications of the partition and insulation^13^,^14^. According to the above principles, in the paper, we proposed to prepare a cellular lightweight material which integrated the advantages of MPC (fast hardening, high early strength, and better durability) with the feature of foam concrete (uniform pore distribution). This new material might allow the potential application in construction and absorption material fields, such as thermal insulation materials. However, actually, the porous material of MPC was seldom reported because it was difficult to compromise the rapid hardening with the foaming process to achieve the high strength and high porosity simultaneously.

In the paper, a novel porous material, high-porosity magnesia phosphate paste (HPMPP), was prepared with the pre-foaming method, in which the foaming agent was firstly mixed with water, aerated to form foam and then added into the paste. A high content of foam agent was incorporated into the magnesium phosphate paste to form a rapid-hardening porous material with the tailorable high strength and porosity. We studied the effects of the additions of sodium silicate and polypropylene fibers on the strength and porosity of the prepared porous material. Moreover, we explored the mechanism of hydration hardening reaction, the composition, and the pore structure, which determined the mechanical and transport properties of the material.

## Results

### Influences of the ratios of S/So and PF/So on the bulk density

**Figure 1:**
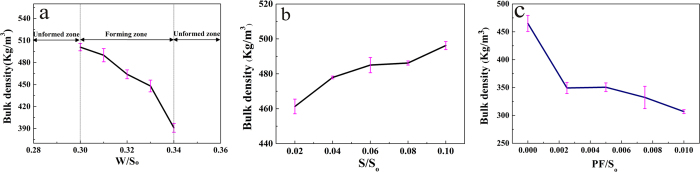
(**a**) Effect of water on the formation of HPMPP and bulk density of HPMPP with different W/S_o_ ratios; (**b**) Effect of the S/S_o_ ratio on bulk density of HPMPP; (**c**) Effect of the PF/S_o_ ratio on bulk density of HPMPP.

As the foam is introduced into the MPC paste, the consistency of the paste is an important factor for HPMPP to get a reasonable density. Figure 1(a) shows the effects of various water contents on the formation process of HPMPP as well as the density variation caused by different W/S_o_ ratios. HPMPP cannot be obtained when the W/S_o_ ratio is below 0.30. The phenomenon can be interpreted in two aspects. Firstly, under the lower consistency, the paste with the lower water content is too stiff to be mixed properly, thus causing the broken bubbles and increasing the damage of the porous structure. Secondly, the lower water content allows the shorter time (less than 30 s) for mixing the paste and poring the paste into the steel molds. When the W/S_o_ ratio is higher than 0.34, it is difficult to form HPMPP and impossible to obtain the appropriate density because the higher water content makes the slurry too thin to maintain the bubbles, thus resulting in the segregation of the foam from the paste^15^,^16^. The density of HPMPP was decreased gradually when the W/S_o_ ratio was changed from 0.30 to 0.34, as shown in Fig. 1(a). When the W/S_o_ ratio was 0.30, the density was about 500.00 ± 5.00 Kg/m^3^. When the W/S_o_ ratio was increased to 0.34, the density reached 390.00 ± 5.00 Kg/m^3^. Figure 1(b) shows the effect of sodium silicate on the density under the W/S_o_ ratio of 0.32. The density of HPMPP increased slowly with the addition of sodium silicate within a reasonable range. Figure 1(c) shows the variation of density with the content of polypropylene fibers under the W/S_o_ ratio of 0.32 and the sodium silicate to solids ratio (S/S_o_) of 0.06. The density decreased significantly when the addition of polypropylene fibers was increased. Under the S/S_o_ ratio of 0.06, the density obtained under the ratio of polypropylene fibers to solids (PF/S_o_) of 0.0025 was lower than that obtained without PF because the added fibers were randomly distributed in the spatial structure to replace magnesium phosphate and act as the bridge in the framework. With the increase in the addition of PF, the density showed a sharp decline and even became lower than that obtained without polypropylene fibers because the excessive dosage of polypropylene fibers greatly decreased the solid content as well as the compressive strength.

### Influences of the ratios of S/So and PF/So on the compressive strength and apparent porosity

**Figure 2:**
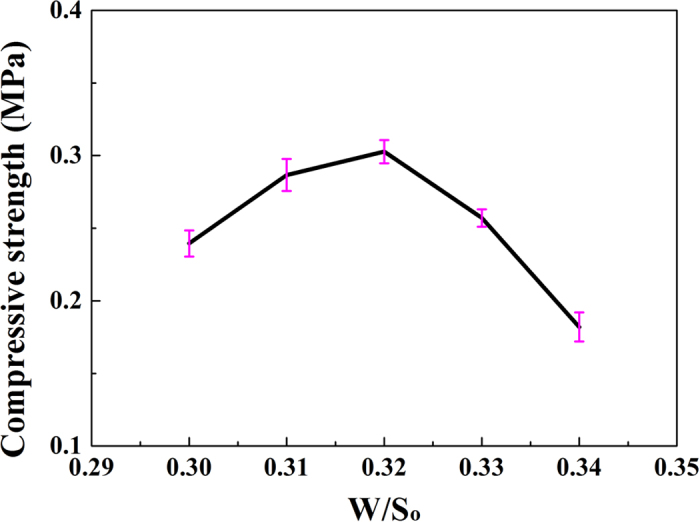
Effect of the W/S_o_ ratio on compressive strength of HPMPP.

The relationship between the W/S_o_ ratio and the compressive strength is shown in Fig. 2. The compressive strength of HPMPP was improved when the W/S_o_ ratio was increased from 0.30 to 0.32 under the ratio of S/S_o_ of 0.04. Under the W/S_o_ ratio of 0.32, the compressive strength was about 0.30 ± 0.05 MPa. When the W/S_o_ ratio increased to 0.34, the compressive strength decreased to 0.18 ± 0.05 MPa. When the W/S_o_ ratio was below 0.34, it was difficult to form HPMPP.

**Figure 3:**
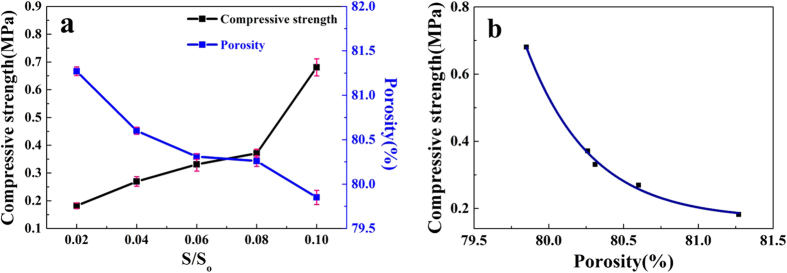
(**a**) Effect of the S/S_o_ ratio on compressive strength and porosity of HPMPP; (**b**) Relationship between compressive strength and porosity with under different S/S_o_ ratios.

The compressive strength of HPMPP was decreased to 0.1 MPa after foam was added into MgO-NH_4_H_2_PO_4_-H_2_O ternary system. In order to increase the compressive strength, sodium silicate was introduced into HPMPP paste. Although all the specimens had a similar setting time, various HPMPP pastes prepared with different S/S_o_ ratios showed the significant differences in physical properties. The comparison results of compressive strength and apparent porosity of various HPMPP pastes obtained after 28-day curing are shown in Fig. 3(a). When the ratio of S/S_o_ was increased, the compressive strength was increased and the apparent porosity was decreased slowly. When the S/S_o_ ratio was increased from 0.02 to 0.08, the compressive strength of the paste was increased slowly. When the S/S_o_ ratio was increased to 0.10, the compressive strength reached its highest value, 0.68 ± 0.05 MPa, which was almost 70% more than the value obtained under the S/S_o_ ratio of 0.08. Besides, the addition of sodium silicate also significantly increased the apparent porosity, which reached its highest value of 81.27% when the S/S_o_ ratio was 0.02.

A higher physical strength depended on the crystal growth, the improved formulation, and the efficient constituent consumption. The chemical reactions of the MgO-NH_4_H_2_PO_4_-H_2_O ternary system started when NH_4_H_2_PO_4_ contacted with water in the mixing stage. The chemical reaction of the hexahydrate formation is:


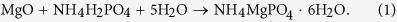


The above reaction also implies that the reactants are completely consumed providing that the theoretical molar ratio of M/P is 1:1^17^,^18^. However, the raw materials, especially the high-porosity material, could not be consumed completely during the rapid reaction. Therefore, the compressive strength of HPMPP is far less than that of dense magnesium phosphate cement paste because the compressive strength of struvite is low. When the S/S_o_ ratio is increased to 0.10, the compressive strength shows a sharp increase because excessive sodium silicate fills the pores and enhances pore wall strength and the apparent porosity is decreased to 79.85%. The relationship between compressive strength (F) and apparent porosity (P_A_) under different S/S_o_ ratios shown in Fig. 3(b) is simulated according to Eq. (2):


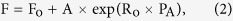


where F_o_, A, and R_o_ are the constants and respectively equal to 0.18892, 6.51922, and −2.65632.

**Figure 4:**
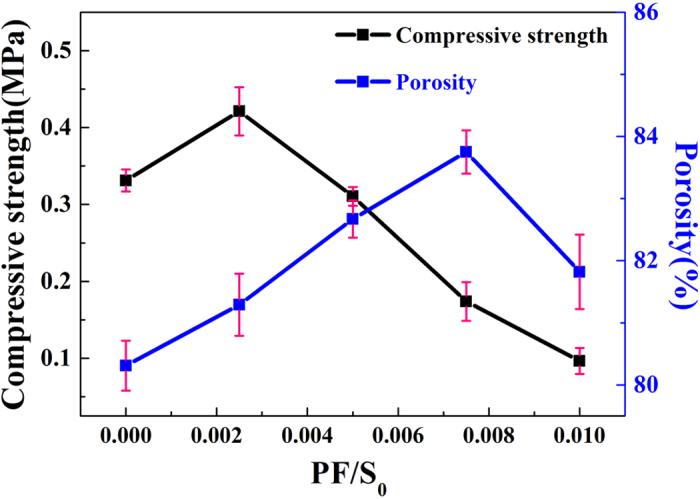
Effect of the PF/S_o_ ratio on compressive strength and porosity of HPMPP.

In order to further improve the compressive strength and porosity, polypropylene fibers were added into the HPMPP paste. The effects of PF on the compressive strength and porosity are shown in Fig. 4. Under the W/S_o_ ratio of 0.32 and the S/S_o_ ratio of 0.06, the compressive strength of HPMPP increases from 0.33 ± 0.05 MPa to 0.42 ± 0.05 MPa when the PF/S_o_ ratio increases from 0 to 0.0025. Then the compressive strength decreases slowly with the increasing of the PF/S_o_ ratio, because of the destruction of pore wall by excessive polypropylene fibers.

**Figure 5:**
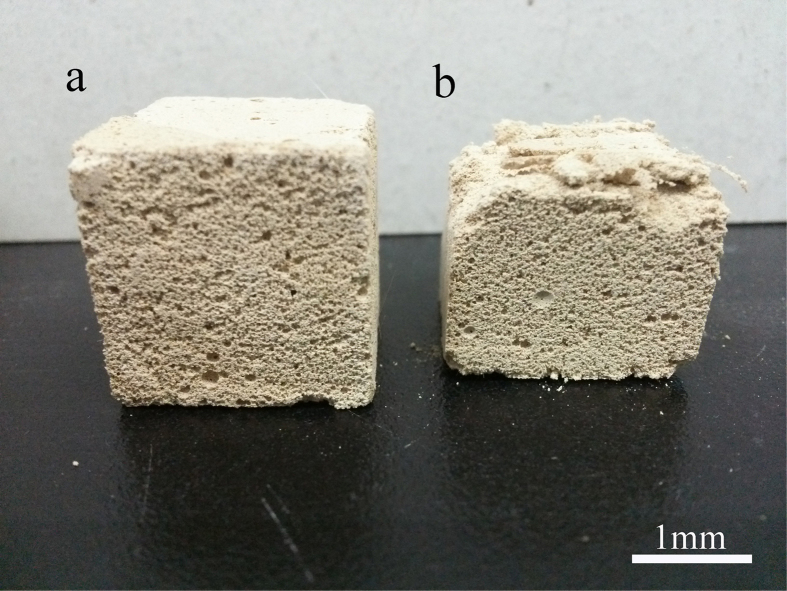
Effect of 1-MPa pressure on the shape change of HPMPP with the PF/S_o_ ratio of 0.0025. (**a**) before applying the pressure; (**b**) after applying the pressure.

**Figure 6:**
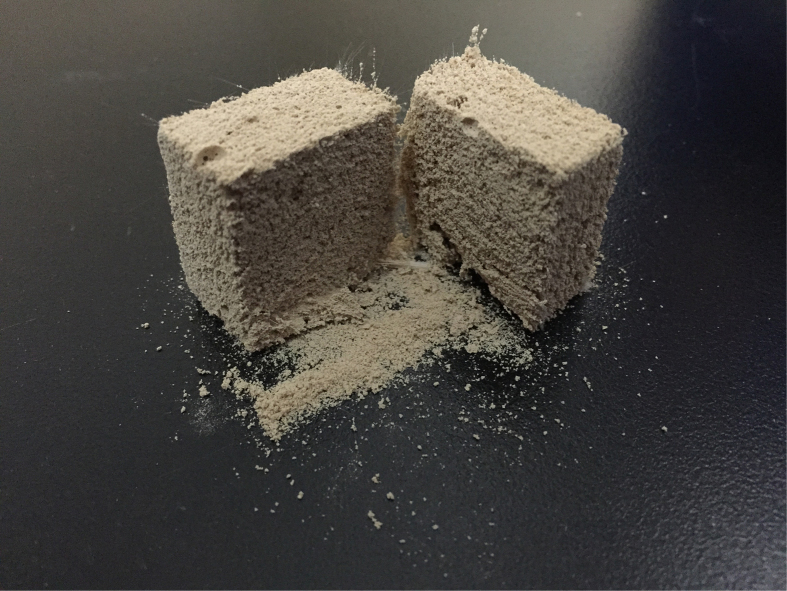
Effect of excess polypropylene fibers on the porous structure of HPMPP with the PF/S_o_ ratio of 0.01.

As shown in Fig. 4, when the PF/S_o_ ratio increases from 0 to 0.0075, the porosity increases from 80.31% to 83.75%, and then a sharp decrease appeared with the continuous increase of polypropylene fibers. A reasonable explanation is that the polypropylene fibers play two roles in HPMPP system: support and destruction. When a small amount of polypropylene fibers are introduced into the paste, the compressive strength of the matrix is enhanced because polypropylene fibers replace the magnesium phosphate and act as the supporting framework of the matrix. As shown in Fig. 5(a,b), HPMPP with PF/S_o_ of 0.0025 was destroyed partly during the process of compression. When the pressure of 1 MPa was applied on the specimen, the upper part of the specimen was damaged while the lower part was still in the perfect conditions (Fig. 5(b)). The results indicate that HPMPP will not collapse when it is extruded by external forces which will help HPMPP to make a difference in practical application. However, the excessive polypropylene fibers (such as PF/S_o_ of 0.01) would results in the porosity decrease. The reason for the significant decrease in the porosity is that the breaking of bubbles by excessive polypropylene fibers leads to the increase of the destruction degree of pore wall. As shown in Fig. 6, a large amount of loose powder dropped out when the sample of HPMPP with PF/S_o_ of 0.01 was cut off and the structures were destroyed. In consequence, the porosity is rather changeable with the addition of polypropylene fibers and the regulation for the change of porosity is unstable. However, according to the report by Mahalingam *et al.*^19^, the destructive effect of polypropylene fibers on porous structure may be decreased by more stable bubbles. Thus, HPMPP of the higher strength and porosity may be achieved by increasing the stability of bubbles in the future work.

### Microstructure analysis

**Figure 7:**
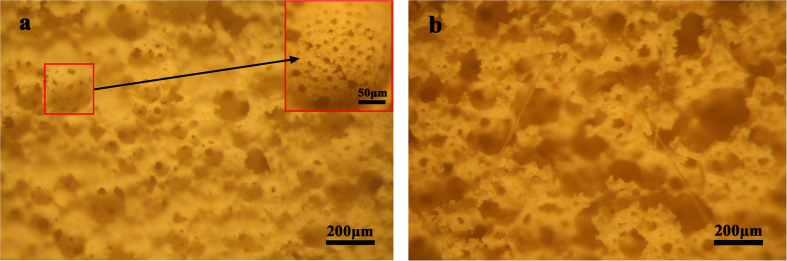
X-ray diffractograms of the HPMPP.

Figure 7 presents the X-ray diffraction spectra of HPMPP under the magnesia to phosphate ratio (M/P) of 4 and the W/S_o_ ratio of 0.32. In the XRD pattern, the peaks of NH_4_MgPO_4_·6H_2_O and MgO were observed. The absence of NH_4_H_2_PO_4_ diffraction patterns indicated that NH_4_H_2_PO_4_ particles were consumed in the hydrated reaction. The diffraction peaks of NH_4_MgPO_4_·6H_2_O illustrated that the HPMPP paste experienced a high degree of the hydrated reaction and that the hydration product was relatively pure. Besides, the diffraction peaks of MgO were also observed in the XRD pattern, and the diffraction intensity was still high even after 28 days, indicating that much unreacted magnesia existed in the HPMPP. Excessive MgO was used in this experiment to allow the high strength of HPMPP.

**Figure 8:**
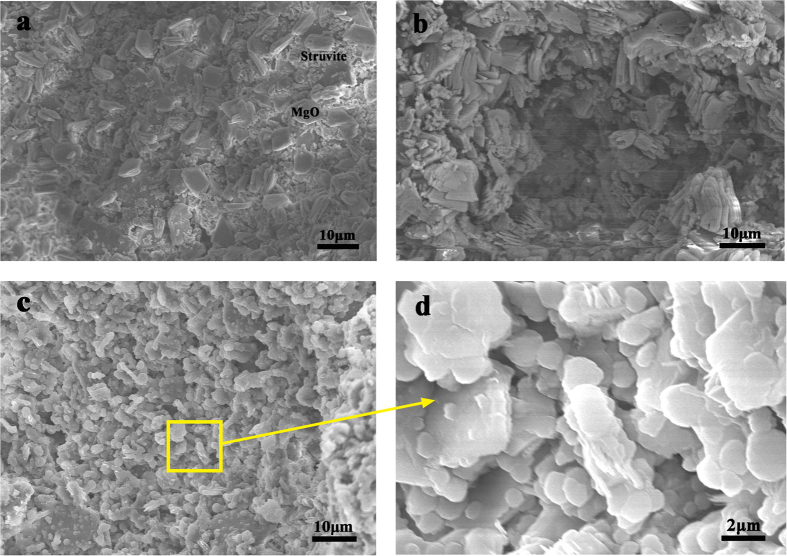
Light optical pictures of HPMPP (**a**) without PF, (**b**) with PF/S_o_ of 0.0025.

The optical microscopic photos of HPMPP are shown in Fig. 8. It is can be seen from Fig. 8(a) that the majority of the pores are in the uniform size and that the average pore diameter is about 180 μm. In addition, a few bigger pores are observed due to the possibility of merging and overlapping of pores. However, the pore distribution is relatively homogenous. The pore wall is thin, thus allowing a low bulk density and a high porosity. As shown in Fig. 8(b), polypropylene fibers acted as the internal support of the porous material. Therefore, although the addition of polypropylene fibers partly destroyed the structure of pores, the compressive strength was increased above 0.1 MPa. Moreover, the existence of polypropylene fibers also decreased the porosity of HPMPP from 80.31% to 83.75%.

**Figure 9:**
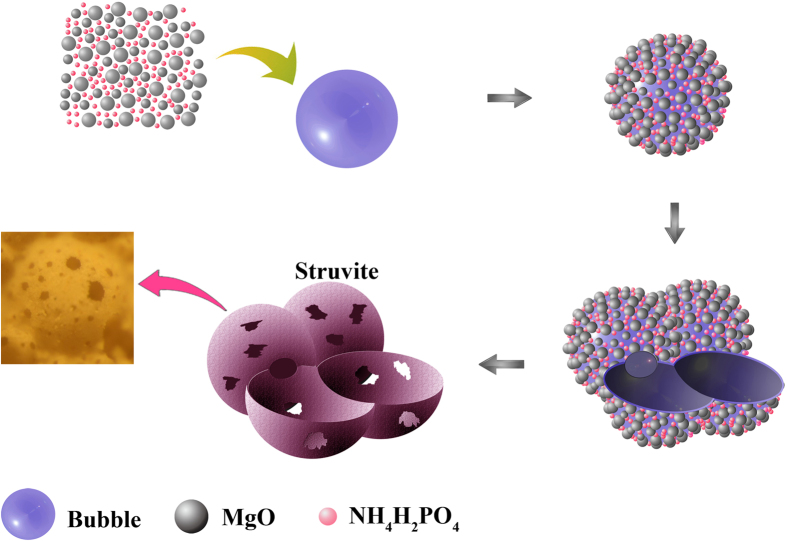
SEM of HPMPP with different S/S_o_ ratios (**a**) 0.02, (**b**) 0.06, (**c**,**d**) 0.10.

Figure 9 shows the microstructure of the HPMPP products prepared under various S/S_o_ ratios. As shown in Fig. 9(a), granulous crystals are formed under the S/S_o_ ratio of 0.02. The granulous microstructure of struvite crystals was consistent with the reports by Fei Qiao and Soude´e^20^,^21^. The tabular magnesia residues were surrounded by struvite crystals and scattered inside HPMPP and the loose structure led to the low strength. With the increase in the S/S_o_ ratio, well-crystallized hydrates wew observed (Fig. 9(b)). The struvite crystals were closely stacked around magnesia grains. Struvite crystals coated on magnesia grains formed a cementitious matrix to bond magnesia grains. At the same time, magnesia residues still existed. Compared with Fig. 9(a–c) shows the more closely packed structure of MCP formed after the addition of sodium silicate. Continuous tabular crystals are identified with an unordered morphology. Under a higher magnification (Fig. 9(d)), these crystals with irregular shapes are slightly different from struvite. Actually, previous work has revealed the possibility of the existence of silico-phosphate bonds^21^,^22^. Therefore, it is believed that amorphous silica in sodium silicate is likely to participate in the chemical reaction to form strong bonds, which is not confirmed by the detection methods adopted in the paper. Considering the size and structural form of the crystals, the reaction product is supposed to be struvite crystals containing the silico-phosphate bonds. Besides, it possesses the highest mechanical strength among various pastes, which can be confirmed by the more compacted microstructure as shown in Fig. 9(c). The product may be responsible for the high porosity and high strength observed in the HPMPP specimens with sodium silicate.

## Discussion

The system of MgO-NH_4_H_2_PO_4_-H_2_O is usually considered as the ternary system^23^ and the dissociation of raw materials is an indispensable part of the solidifying process of HPMPP. The formation of the ternary system starts from the dissolution of NH_4_H_2_PO_4_ when it contacts with water during the mixing stage, and then releases NH_4_^+^, H^+^, PO_4_^3−^, HPO_4_^2−^, and H_2_PO_4_^−^ ions to produce an acidic environment which will induce the dissociation of MgO. MgO releases Mg^2+^ and OH^−^ under the actions of H_2_O and H^+^. The dissociation process of MgO is provided as follows^24^,^25^:


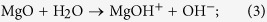



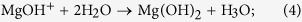



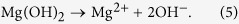


Then the octahedral complex Mg(H_2_O)_6_^2+^ was formed with Mg^2+^ and six H_2_O molecules and coated on the surface MgO. Subsequently, the crystalline product, NH_4_MgPO_4_·6H_2_O (struvite), was formed with Mg(H_2_O)_6_^2+^, PO_4_^3−^, and NH_4_^+^. Then a struvite network was formed under the role of hydrogen bond. However, when the foam was introduced into the MPC paste, the content of water surrounding MgO particles decreased, thus reducing the degree of reaction. Then, the strength of HPMPP was decreased due to the reduction of struvite network.

**Figure 10:**
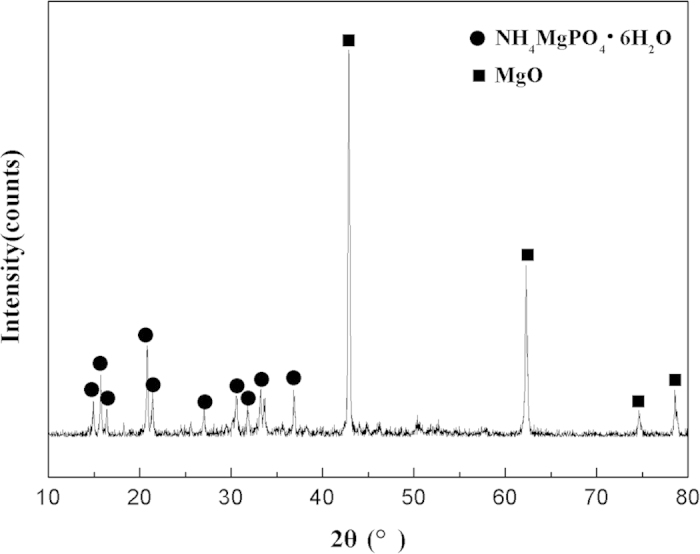
Simulated microstructural evolution of the HPMPP paste.

Figure 10 shows the simulated microstructure evolution of the HPMPP paste with the M/P of 4. When magnesia and phosphate powders are blended with the mixture of water, sodium silicate and borax, the phosphate is dissolved immediately to form an acidic aqueous medium and basic magnesia is dissolved to release magnesium ions into solution simultaneously. Since the reaction between magnesium ions and phosphate is very fast, the mixture is poured into bubbles quickly. Then, magnesia and phosphate are absorbed on the surface of bubbles and struvite is formed rapidly along with the strong hydration reaction. Then the porous structure can be achieved after breaking bubbles due to water reduction. However, this is an ideal reaction status. Generally, the excessive magnesia cannot be consumed completely in the reaction and struvite gradually grows around magnesia particles. Then struvite is combined with MgO particles one by one and coated on the surface of the bubbles. The more MgO particles combined with struvite leads to the thicker pore wall as well as the higher strength of HPMPP. After the reaction, an open structure is finally formed, as shown in Fig. 10.

The relationship between strength and density is also important for HPMPP and foam concrete. When the density is given, the strength can be increased by changing the constituent materials. The foam consumption also depends on the constituent materials. Therefore, the rapid-hardening magnesia phosphate paste with high strength and porosity can be achieved by means of controlling the stoichiometric proportions of raw materials. Moreover, its strength and porosity can be tailored.

## Methods

### Raw materials

Like MPC, high-porosity magnesia phosphate paste (HPMPP) was prepared from a mixture of dead burned magnesia oxide, chemical grade ammonium dihydrogen phosphate and borax. In addition, various proportions of sodium silicate, 9-mm long DULIE PF5 polypropylene fibers, and foam agent were adopted in this study. The dead burned magnesia powder calcined above 1500 °C had the average particle size of about 10 μm (Haicheng, Liaoning Province, China). The magnesia particle size distribution was analyzed by a laser particle size analyzer (Mastersizer 2000, Malvern Instruments Ltd., UK). The particle morphology was observed by scanning electron microscopy (SEM, Hitachi, S4800). The chemical composition of the dead burned magnesia was characterized by an X-ray fluorescence spectrometer (XRF, JEOL JSX-3201Z), as shown in Table 1. Moreover, 9-mm long polypropylene fibers (Beijing Ronel Engineering materials Co., LTD) were added to enhance the shear behaviors of the prepared high-porosity magnesia phosphate paste. The addition of fibers can mitigate brittleness and reduce weight and cost.

### Experimental method and procedure

The fresh pastes of basic HPMPP were prepared according to the following procedure. MgO, NH_4_H_2_PO_4_, foam, potable borax, sodium silicate, and water was firstly mixed for about 2 min and then poured into steel molds (20 × 20 × 20 mm^3^). The mixing materials of various pastes are different in the contents of sodium silicate and water. When the pastes were poured into the molds, gentle vibration was performed to remove large bubbles. The specimens were removed from the mold in 7 days after casting and then cured in a standard curing chamber at 20 ± 1 °C and under the relative humidity of 90 ± 5% for another 21 days. Polypropylene fibers were added into the paste according to different proportions to enhance the strength of the rapid-hardening materials. The mixing proportions and other details of the raw materials of the HPMPP specimens are provided in Table 2.

### Characterization

In the tests of compressive strength, the cubic specimens (20 × 20 × 20 mm^3^) were tested by an electromechanical universal testing machine according to the loading rate of 1 kN per second. The microstructure was examined under an optical microscopy (equipped with a digital image analysis system) and scanning electron microscope (SEM, Hitahi, S4800). The phase compositions of the specimens were characterized by X-ray diffraction (D’Max-Ra12 kW, Ouyatu, Japan) and the diffraction spectrum was obtained in the range of 2*θ* (10°–80°) at the scanning rate of 2°/min. The apparent porosity and density were determined with the technique based on the Archimedes principle. The specimens were weighed after 12-h drying in an oven (M_1_). The specimens were then pumped to form vacuum and then water was injected into the specimens. The immersed mass (M_2_) and the wet mass (M_3_) were determined after the specimens were immersed in water for 1 h and fully saturated. Therefore, the apparent porosity (P_A_) and the bulk density (D_A_) were respectively calculated according to Eqs (6) and (7)^3^,^26^:


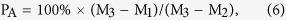



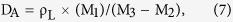


where ρ_L_ is the water density (ρ_L_ = 1000 Kg/m^3^ at 25 °C).

## Additional Information

**How to cite this article**: Liu, L.-J. *et al.* Tailoring the strength and porosity of rapid-hardening magnesia phosphate paste via the pre-foaming method. *Sci. Rep.* **5**, 13016; doi: 10.1038/srep13016 (2015).

## Figures and Tables

**Table 1:** Chemical compositions of magnesia (wt.%).

SiO_2_	TiO_2_	Al_2_O_3_	TFe_2_O_3_	MnO	MgO	CaO	Na_2_O	K_2_O	P_2_O_5_	Loss	Total
3.25	0.03	0.94	1.06	0.06	91.18	1.81	0.04	0.02	0.09	1.02	99.50

**Table 2:** Mixing proportions of the magnesium phosphate pastes.

Materials	Weight ratios
P/M	0.25
B/M	0.075
W/S_o_	0.30 ~ 0.34
S/S_o_	0.02 ~ 0.10
PF/S_o_	0 ~ 0.01

P-phosphate, B-borax, M-magnesia, W-water, S-sodium silicate, PF-polypropylene fibers, S_o_-solids consisting of magnesia and phosphate.
