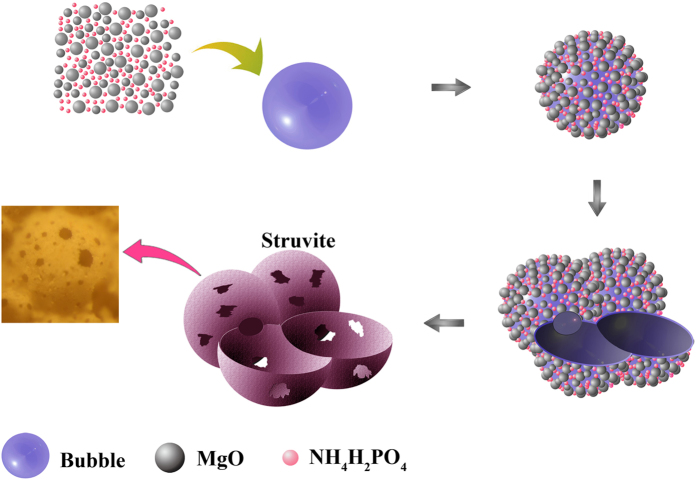# Corrigendum: Tailoring the strength and porosity of rapid-hardening magnesia phosphate paste via the pre-foaming method

**DOI:** 10.1038/srep14898

**Published:** 2015-10-08

**Authors:** Li-Jie Liu, Jin-Hong Li, Xiang Wang, Ting-Ting Qian, Xiao-Hui Li

Scientific Reports 5: Article number: 13016; 10.1038/srep13016 published online: 08 13 2015; updated: 10 08 2015.

This Article contains an error in the order of the Figures. Figs 7, 8, 9 and 10 were published as Figs 10, 7, 8 and 9 respectively. The correct Figs 7–10 appear below as Figs 1, 2, 3, 4 respectively. The Figure legends are correct.

## Figures and Tables

**Figure 1:**
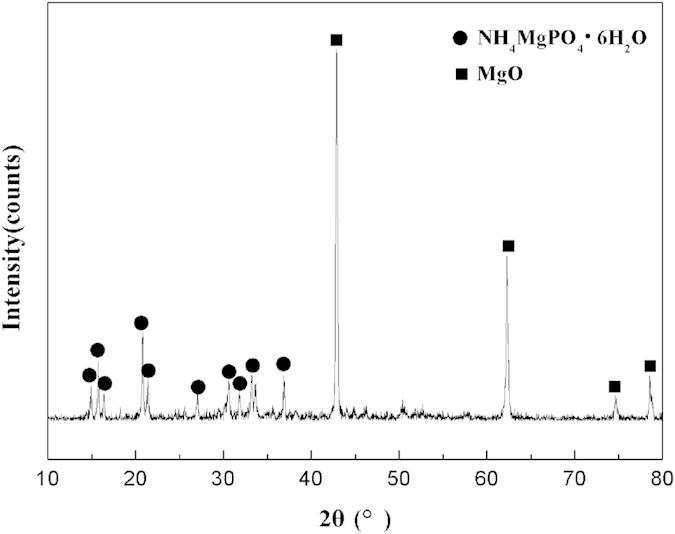


**Figure 2:**
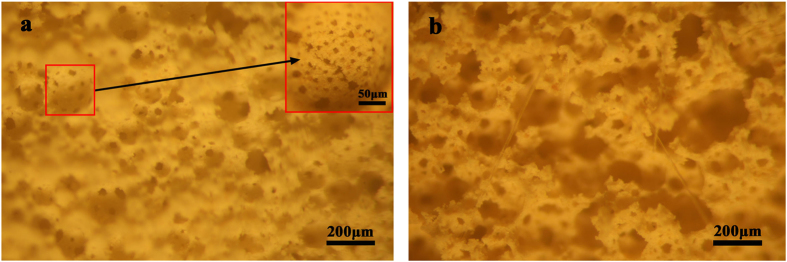


**Figure 3:**
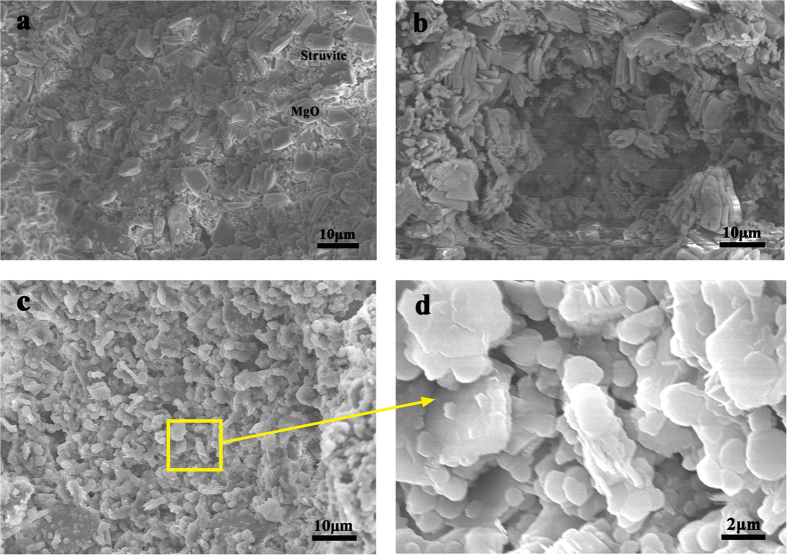


**Figure 4:**